# A Comparative Study of Theoretical Graph Models for Characterizing Structural Networks of Human Brain

**DOI:** 10.1155/2013/201735

**Published:** 2013-11-27

**Authors:** Xiaojin Li, Xintao Hu, Changfeng Jin, Junwei Han, Tianming Liu, Lei Guo, Wei Hao, Lingjiang Li

**Affiliations:** ^1^School of Automation, Northwestern Polytechnical University, Xi'an 710071, China; ^2^Department of Psychiatry, The Mental Health Institute, The Second Xiangya Hospital, Central South University, Changsha, China; ^3^Department of Computer Science and Bioimaging Research Center, University of Georgia, Athens, GA 30602, USA

## Abstract

Previous studies have investigated both structural and functional brain networks via graph-theoretical methods. However, there is an important issue that has not been adequately discussed before: what is the optimal theoretical graph model for describing the structural networks of human brain? In this paper, we perform a comparative study to address this problem. Firstly, large-scale cortical regions of interest (ROIs) are localized by recently developed and validated brain reference system named Dense Individualized Common Connectivity-based Cortical Landmarks (DICCCOL) to address the limitations in the identification of the brain network ROIs in previous studies. Then, we construct structural brain networks based on diffusion tensor imaging (DTI) data. Afterwards, the global and local graph properties of the constructed structural brain networks are measured using the state-of-the-art graph analysis algorithms and tools and are further compared with seven popular theoretical graph models. In addition, we compare the topological properties between two graph models, namely, stickiness-index-based model (STICKY) and scale-free gene duplication model (SF-GD), that have higher similarity with the real structural brain networks in terms of global and local graph properties. Our experimental results suggest that among the seven theoretical graph models compared in this study, STICKY and SF-GD models have better performances in characterizing the structural human brain network.

## 1. Introduction

The human brain is intrinsically organized into distinct large-scale functional networks, and cognitive functions arise from the dynamic interactions of distributed brain areas operating in these networks [1]. New advances in neuroimaging techniques have shown the possibility of systematic exploring the human brain formal complex network perspective. Graph theory provides a theoretical framework in which the topological properties of the brain networks can be examined such as centrality, clustering, efficiency, hierarchy, modularity, robustness, small-worldness, and synchronizability [2], and it can reveal important information about both the global and local organizations of the human brain networks. The improved characterization of brain networks achieved via graph-theoretical methods provides not only parsimonious accounts of normal cognitive processes [3], but also novel insights into psychiatric and neurological disorders such as Alzheimer's disease [4, 5], multiple sclerosis [6], and attention-deficit disorder [7].

Many complex systems show remarkably similar macroscopic behaviors despite profound differences in the microscopic details of the elements of each system or their mechanisms of interaction [2]. In this paper, we focus on an important issue that has not been adequately addressed before: what graph models can best possibly describe the structures of brain networks. In this way, the model provides a possible explanation for a key challenge for systems neuroscience: how to understand the complex network organization of the brain on the basis of neuroimaging data [8]. For instance, previous studies have demonstrated the small-worldness [9] and scale free properties of human brain networks [10].

In brain network studies, the methods for identifying regions of interest (ROIs), that is, the network nodes, can be generally classified into four categories. The first group is manual labeling by experts based on experience and domain knowledge. This method is widely used; however, it may not be reproducible due to both intersubject and intrasubject variations [11]. The second group is data-driven methods, which clusters ROIs from the brain image itself [12, 13]. However, it might be sensitive to the clustering parameters used in many data-driven approaches. The third group of methods identifies activated brain regions as ROIs by task-based functional magnetic resonance imaging (task-based fMRI), and it is regarded as the benchmark approach for ROI identification. However, task-based fMRI itself has limitations such as being time-consuming and expensive. Additionally, different patterns may be shown in group-based activation maps from an individual's activation map [14]. In short, it remains quite challenging to accurately localize ROIs for each individual by using standard analysis of task-based fMRI data [15]. The last group methods are cortical parcellation based on image/surface registration, whose limitations have been comprehensively discussed in [16, 17].

In this paper, we apply our recently developed brain reference system, named Dense Individualized and Common Connectivity-based Cortical Landmarks (DICCCOL) [18] which discovers 358 consistent and corresponding ROIs across subjects based on diffusion tensor imaging (DTI) data, to localize the ROIs for each participated subject. DICCCOL possess intrinsically established structural and functional correspondences (universal), while their locations and sizes are determined in each individual's space (individualized). With the identified brain ROIs, the structural brain network is constructed for each subject based on the corresponding DTI data. Then, through the large network analysis tool GraphCrunch2 [19], we evaluate the fitness of seven popular theoretical graph models for describing the real brain network by measuring both the global and local graph properties of the constructed brain networks and compare them with those graph models. The graph models are as follows: (1) Erdős-Rényi random graph (ER) [20]; (2) Erdős-Rényi random graph with the same degree distribution as the input data (ER-DD) [19, 21]; (3) geometric random graph (GEO) [22, 23]; (4) geometric gene duplication model (GEO-GD) [24]; (5) scale-free Barabási-Albert preferential attachment model (SF) [25]; (6) scale-free gene duplication model (SF-GD) [26]; (7) stickiness-index-based model (STICKY) [27]. Those graph models will be explained in detail in Section 2.

Our experimental results suggest that the SF-GD model fits the real brain network the best in terms of global and local graph properties. We also demonstrate that the real brain network also has the STICKY property. In summary, the SF-GD model, combined with its STICKY property, can best describe graph properties of the real brain network and also can indirectly describe the mechanism of structural network from biological properties. Importantly, the results are consistent across populations.

## 2. Materials and Methods

### 2.1. Data Acquisition and Preprocessing

DTI datasets for 104 healthy subjects including three age groups of adolescents (28), young adults (53), and elderly normal brains (23) were acquired on a 3T GE Signal magnetic resonance imaging (MRI) scanner. Acquisition parameters for the scans were as follows: 256 × 256 matrix, 3 mm slice thickness, 240 mm^2^ field of view (FOV), 50 slices, 15 diffusion weighted imaging (DWI) volumes, and *B* value = 1000. The preprocessing of DTI data included brain skull removal, motion correction, and eddy current correction [28]. After pre-processing, fiber tracking was reconstructed via MEDINRIA [29]. Then, The grey matter (GM)/white matter (WM) cortical surface was reconstructed according to the brain tissue segmentation map based on the DTI data [30].

### 2.2. Structural Brain Network Construction

The structural brain network of each subject is represented as an unweighted undirected graph *G* = (*V*, *E*) in which *V* = {*v*_*i*_, *i* = 1, 2, 3, …, *N*} is the set of nodes and *E* = {*e*_*ij*_, *i*, *j* = 1, 2, 3, …, *N*} is the set of edges, where *N* is the number of nodes in the network. The nodes in *V* are identified via DICCCOL system [18], and the elements in *E* are measured for each ROI pair independently. Specifically, DICCCOL employs a novel data-driven strategy to discover, from DTI datasets, dense and common cortical landmarks [18]. The basic idea is that we optimize the localizations of each DICCCOL landmark in individual subjects by maximizing the groupwise consistency of their white matter fiber connectivity patterns. We obtain 358 DICCCOL ROIs for each subject and regard them as network nodes. White matter fibers obtained via the deterministic DTI tractography are projected onto the reconstructed cortical surface and the number of fibers connecting the ROI pair is used to measure the structural connectivity between the ROI pairs. Thus, a structural connectivity matrix is obtained for each subject. A predefined threshold *T* is applied on the connectivity matrix to remove the noise and errors in fiber tracking. Note that *T* is the same for all the subjects. After thresholding the structural connectivity matrix, we obtained the adjacency matrix *A*_*N*×*N*_ for each subject. In the adjacency matrix, two nodes *v*_*i*_ and *v*_*j*_ are connected if *a*_*ij*_ = 1; otherwise, *a*_*ij*_ = 0. The self-loops are currently ignored in the constructed structural brain networks.

**Figure 1:**
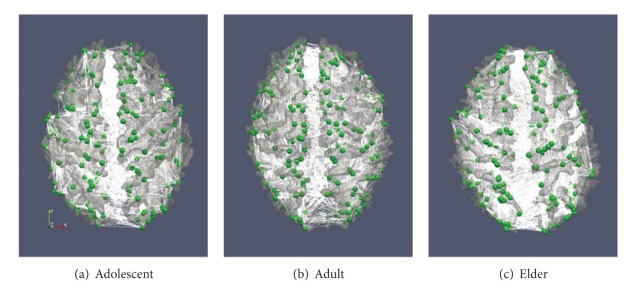
Examples of structural brain networks in different groups. (a) Adolescent group. (b) Adult group. (c) Elder group. The nodes are represented by green spheres and the edges are represented by white lines. The constructed structural brain networks are overlaid on the corresponding cortical surfaces reconstructed from DTI data.

Following the procedure described above, we constructed structural brain networks for 28 adolescents, 53 adults and 23 elders.  Figure 1 shows exemplar brain networks in different groups.

### 2.3. Graph Models

In this study, we compared the topological properties of the constructed structural brain networks with those of 7 typical graph models via GraphCrunch2 [19], a tool for complex network analysis. GraphCrunch2 implements the following network models: (1) Erdős-Rényi random graph model (ER): ER graph is generated by using the Library of Efficient Data Types and Algorithms- (LEDA-) based random graph generator [19]. ER graph can provide a rigorous definition of what it means for a property to hold for almost all graphs, or be used in the probabilistic method to prove the existence of graphs satisfying various properties [20]. (2) Erdős-Rényi random graph with the same degree distribution as the input data (ER-DD): ER-DD graph is generated by using the “stubs method” [19]. In brief, according to the degree distribution of the real-world network being modeled, the number of “stubs” (to be filled by edges) is assigned to each node in the model network. After that, edges are created between randomly picked pairs of nodes. At last, the number of “stubs” left available at the corresponding “end-nodes” of the edge is decreased by one [19, 21]. (3) Geometric random graph model (GEO): in GEO graph, nodes correspond to uniformly randomly distributed points in a metric space and if the corresponding points are close enough in the metric space, according to some distance norm, the edges are created between pairs of nodes [22, 23]. (4) Geometric gene duplication model (GEO-GD): GEO-GD graph is the extension of geometric random graph, in which the principles of gene duplications and mutations are incorporated [19]. Every model determines the principle by which the network is grown from a small seed network and adds new nodes intended to model gene duplications and mutations [24]. (5) Scale-free Barabási-Albert preferential attachment model (SF) [25]: the most important characteristic in a scale-free network is the relative commonness of vertices with a degree that greatly exceeds the average. The highest-degree nodes are named “hubs.” The scale-free property strongly correlates with the robustness of network. The hierarchy allows for a fault-tolerant behavior [26]. (6) Scale-free gene duplication model (SF-GD): SF-GD model is an evolution of SF model. The tolerance of SF-GD to damage is determined by the scale-free nature of its multifractal distribution. However, they present novel properties: multifractal features are inherited in a model of growing networks [26]. (7) Stickiness-index-based model (STICKY): STICKY graph is based on stickiness indices that summarize node connectivities and the complexities of normalized degree of nodes in networks. The stickiness framework produces a convenient, parameter-free random network [27].

According to the input structural brain network, GraphCrunch2 repeatedly generates a number of instances of the defined graph models and measures the global and local graph properties that infer the similarity/dissimilarity between the input graph and each of the generated model graph instances. In our experiments, the number of repeatedly generated instances is 10. The global and local graph properties implemented in GraphCrunch2 will be introduced in the next section.

### 2.4. Topological Properties of Graphs

We calculated both global and local topological graph properties for the constructed structural brain networks and the 7 graph models. Statistical analysis was conducted to explore which graph model can best describe the constructed brain networks. In this paper, the following global properties are examined: (1) Pearson correlation coefficients between the degree distributions [31]; (2) average shortest path length difference ratio; (3) average clustering coefficient difference ratio. The local graph properties include the relative graphlet frequency (RGF) distance [23] and the graphlet degree distribution (GDD) agreement [32]. We briefly provide the definitions of these measurements below.

#### 2.4.1. Global Graph Properties

Define the degree *k*_*i*_ of a node *i* as the number of neighbors it has in the network. The topological property of a graph can be obtained in terms of the degree distribution *P*(*k*) [2]. The degree distribution is one of indirect measures that reflect the network robustness to insult [8]. We compute Pearson correlation coefficients between the degree distributions of the graph model and the real brain network as the similarities between them in the aspect of degree distributions. Shortest path length plays an important role in characterizing the internal structure of a graph. Shortest path length of a node pair is defined as the path between two nodes in a graph that the number of its constituent edges is minimized. The average of the shortest path length, also known as characteristic path length, is the mean of the shortest paths over all node pairs [2] and is the most widely used measure to functional integration [8]. The clustering coefficient of node *i* is defined as *C*_*i*_ = 2*E*_neighbor,*i*_/(*k*_*i*_(*k*_*i*_ − 1)), where *E*_neighbor,*i*_ is the number of edges between neighbors of *i*. The average clustering coefficient *C* is the mean of *C*_*i*_ over all nodes [2]. In this paper, we use average shortest path length difference ratio (PathDiff) and average clustering coefficient difference ratio (ClustDiff) to measure the difference between graph models and the real brain networks:

(1) $$\begin{array}{c} \text{PathDiff}=\frac{\left|{\text{Path}}_{\text{model}}-{\text{Path}}_{\text{real}}\right|}{{\text{Path}}_{\text{real}}}\times 100\% \end{array}$$

(2) $$\begin{array}{c} \text{ClustDiff}=\frac{\left|C_{\text{model}}-C_{\text{real}}\right|}{C_{\text{real}}}\times 100\%, \end{array}$$

where Path_model_ and Path_real_ are the average of shortest path of models and real brain networks, respectively and *C*_model_ and *C*_real_ are the average of clustering coefficient of models and real brain networks, respectively. Smaller difference indicates higher similarity between two networks.

#### 2.4.2. Local Graph Properties

In addition to above global properties, we also measured the local graph properties to evaluate the fitness of the graph models to the real brain networks. In this paper, the local graph properties include RGF distance and GDD agreement.

Graphlet degree distribution agreement (GDD agreement) [32] is a similarity measure between topologies of two networks based on graphlet degree vector distributions. GraphCrunch2 computes 2–5 nodes graphlets [19]. By calculating the fitness of each of the 73 GDDs of the networks under comparison, GDD agreement contains 73 similarity constraints. High GDD agreement between two networks indicates that they are similar [19]. In general, GDD agreement is a local heuristic metric for measuring network structure. It imposes 73 highly structured constraints, and thus it increases the chances that two networks are truly similar if they are similar with respect to this measure [32].

Relative graphlet frequency distance (RGF distance) [23] is a measure that compares the frequencies of appearance of all 2 to 5 node graphlets in two networks [19]. Since there are 30 possible graphlets on up to 5 nodes, RGF distance includes 30 similarity constraints by examining the fit of 30 graphlets frequencies between two networks. The similarity between two graphs only depends on the differences between relative frequencies of graphlets. Smaller RGF distance indicates higher similarity between networks [19].

## 3. Results and Discussion

### 3.1. Topological Graph Properties

#### 3.1.1. Global Graph Properties

**Figure 2:**
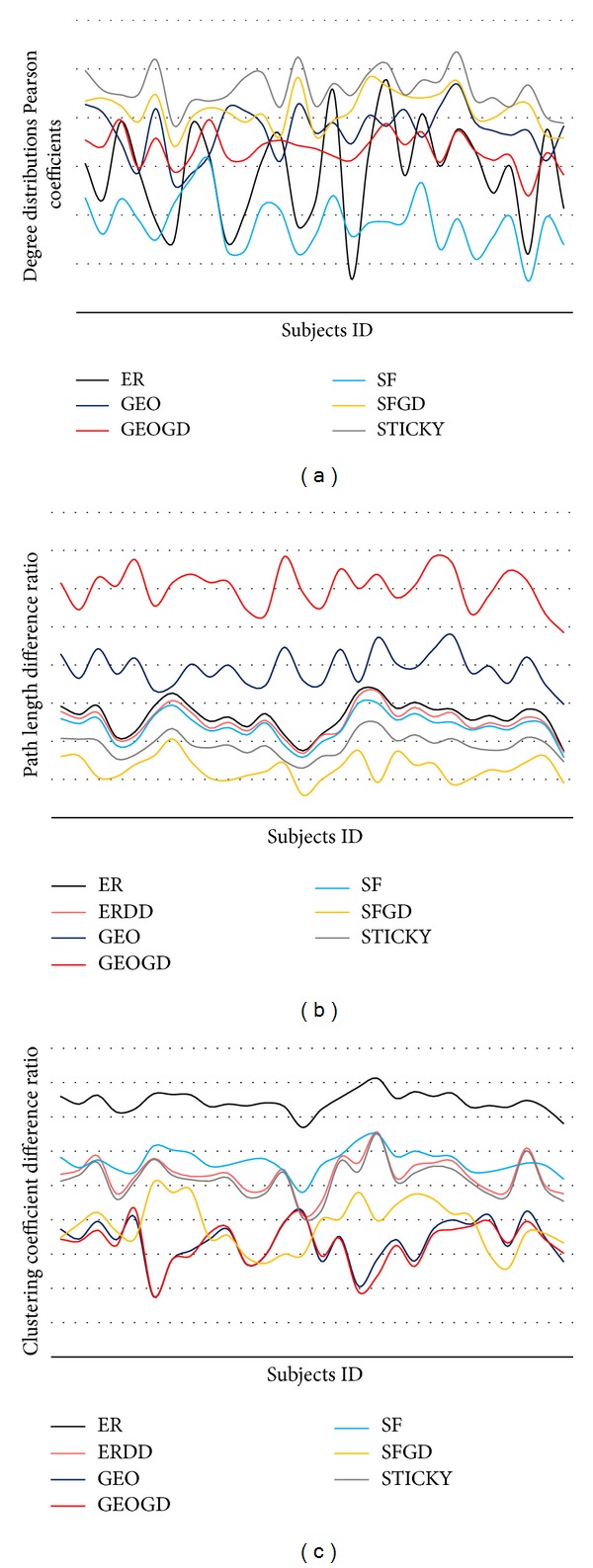
The global graph properties of 7 network models compared with real brain networks (28 subjects). (a) The Pearson correlation coefficients between the degree distributions. Higher value means the graph model can describe the real brain network better. (b) Average shortest path length difference ratio. (c) Average clustering coefficient difference ratio. Lower value means the graph model can describe the real brain network better. The *x*-axis is subject index.

Figure 2(a) shows the Pearson correlation coefficients between the degree distributions of the constructed brain networks and the 7 graph models for 28 subjects in the adolescent group. Note that the ER-DD model needs to be excluded because it has the same degree distribution as the input graph. It is seen that the Pearson correlation of degree distribution is approaching to 1. Figure 2(a) indicates that the STICKY model and SF-GD model have a high correlation with the real structural brain networks. The difference ratio of average shortest path length and average clustering coefficient are shown in Figures 2(b) and 2(c), respectively. It is seen that the SF-GD and STICKY models have lower average shortest path length difference ratio and average clustering coefficient difference ratio.

In general, the comparison of global topological properties between real structural brain networks and the 7 graph models demonstrates the superiority of SF-GD and STICKY graph models in characterizing the structural brain networks. SF-GD and STICKY graph models share common features, namely, “inheritance” and “variance”: when the new nodes are created, the son nodes inherit the connectivities of their parent nodes (“duplication” and “stickiness”). Meanwhile, the son nodes have new connectivities with other nodes which are not linked with their parent nodes “divergence.” These characteristics could indirectly describe the biologically important properties of the mechanism of structural brain network. In the process of brain development, the new neurons are created and contain both functional and structural features. In this way, the specific regions of the brain which have specific function are formed, and the functions of these regions are improved and enhanced, such as the visual association area, motor speech area, and olfactory area. Meanwhile, the new neurons brought additional connectivities with other neurons in different regions; therefore, they enhanced the cooperation and coordination ability of different regions in human brain. The “stickiness” indicates that the important neurons which have large degree in structural brain network will maintain the original functional and structural features during brain development. On the other hand, the SF-GD network shares common features with other scale-free networks, and its tolerance to damage is determined by the scale-free nature of its multifractal distribution. Therefore, it also means that, during development, the brain not only maintains the functional integrity, but also is with increased stability. The SF-GD model has the lowest average shortest path length difference ratio indicates that “divergence” feature enhanced the interactions among different regions during brain development.

#### 3.1.2. Local Graph Properties

**Figure 3:**
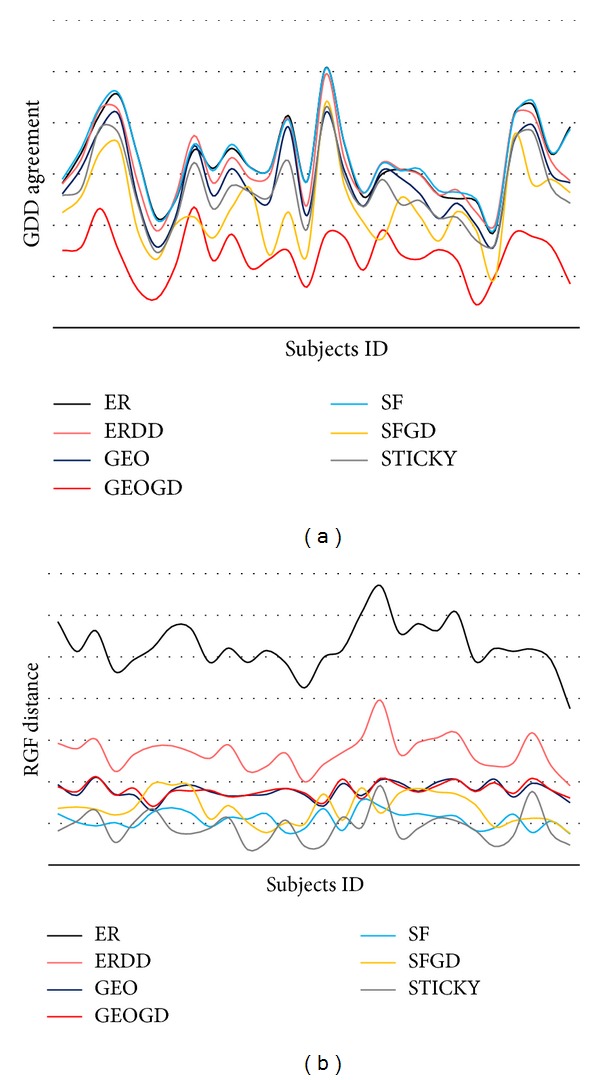
The local graph properties of 7 network models for 28 adolescents. The *x*-axis is subject index. (a) GDD-agreement. Higher value indicates higher similarity. (b) RGF-distance. Higher value indicates lower similarity.

The RGF distance and GDD agreement of the 7 graph models, in comparison with the real structural brain networks for the adolescent group with 28 subjects, are shown in Figures 3(a) and 3(b), respectively. It is seen that the GEO-GD model has the lowest GDD agreements when compared with the real brain networks, and there is no significant difference among the rest of the graph models. It is also shown that STICKY, SF, and SF-GD graph models have lower RGF distance, indicating their superior performance in characterizing the real structural brain networks. It is notable that GraphCrunch2 only calculates 2–5 nodes graphlets of the networks, as mentioned previously. In real brain networks, the connectivity patterns are complex and there exist graphlets with size over 5 nodes. Nevertheless, the local graph properties of RGF distance and GDD agreement demonstrate that STICKY, SF, and SF-GD graph models have higher similarity to the real structural brain networks, especially for the STICKY model. This result reflects that the “stickiness” feature plays an important role in brain development, and it might be the main reason that brain maintains regional and group integrity.

In summary, the results of global and local topological properties indicate that the SF-GD and STICKY graph model fit the brain network data better; that is, the SF-GD and STICKY models have better performance in the description of structural brain networks. Since SF-GD and STICKY are not in conflict [27], we can also regard the stickiness as a property of the real brain network.

### 3.2. SF-GD versus Sticky

**Figure 4:**
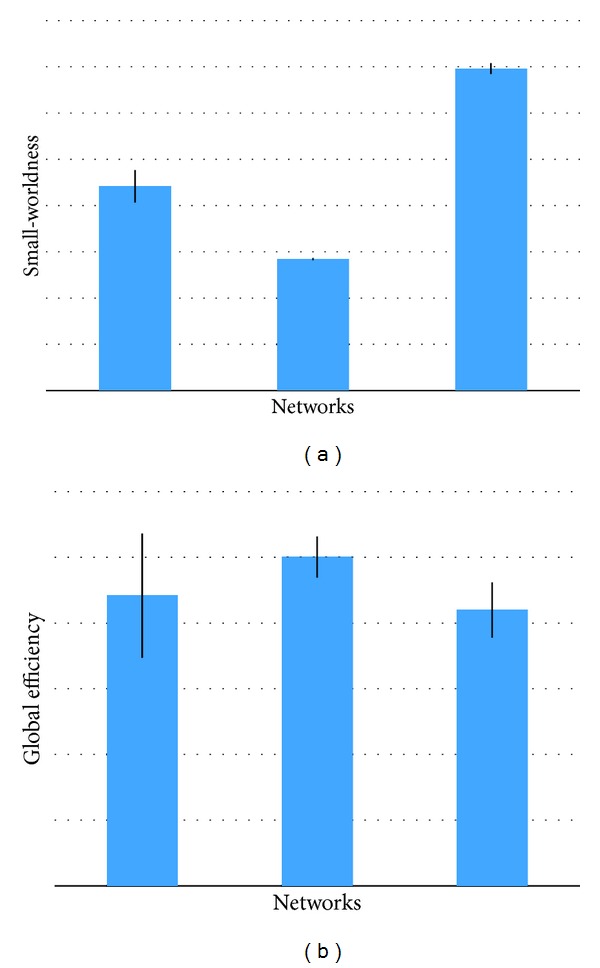
The comparison of small-worldness and global efficiency between SF-GD, STICKY and the real brain networks. (a) Small-worldness. (b) Global efficiency.

The global and local topological properties presented in the previous section indicate that SF-GD and STICKY graph models have comparable performance in describing the real structural brain networks. In this section, we present a further comparison study between SF-GD and STICKY by evaluating two topological properties of complex graph which were not implemented in GraphCrunch2, namely, (1) small-worldness [9]; (2) global efficiency [2]. Small-worldness has been reported as one of the most important properties of both the functional and structural human brain networks in previous studies. Small-worldness networks are defined as networks that are significantly more clustered than random networks, yet have approximately the same characteristic path length as random networks. More generally, small-world networks should be simultaneously highly integrated and segregated [33]. This property is often analyzed by considering the fraction of nodes in the network that have a particular number of connections going into them. The average inverse shortest path length is a related measure known as the global efficiency. Some authors have claimed that the global efficiency may be a superior measure of integration [34]. Unlike the characteristic path length, the global efficiency can be more meaningfully computed on disconnected networks. Zero efficiency corresponds to infinite length of paths between disconnected nodes [8].  Figure 4 shows the mean of small-worldness and global efficiency in the adolescent group. It indicates that the SF-GD model has lower discrepancy compared with the real brain networks in both small-worldness and global efficiency. This result to some extent demonstrates that SF-GD graph model is relatively more suitable in characterizing the human brain networks than STICKY graph model.

### 3.3. Topological Graph Properties across Groups

As a reproducibility study, we compared the topological properties of the brain networks across three different groups (adolescents, adults, and elders). The results are shown in Tables 1, 2, 3, 4, and 5. The trend is almost the same and the difference across the three groups is relatively small. It is also seen that the SF-GD model has higher correlation coefficients of degree distributions, lower average shortest path length difference ratio, and average clustering coefficient difference ratio in all the three groups. It indicates that SF-GD model is the most similar network to the real structural brain networks. The STICKY model also has higher similarity to real brain networks in terms of GDD agreement and RGF distance.

## 4. Conclusion

In this paper, we compared the global and local graph properties, as well as the topological properties of structural human brain networks to 7 representative graph models. The objective is to explore which graph model can best describe structural human brain networks.

Our experimental results demonstrated that SF-GD graph model in general has the best performance in characterizing the structural networks of the human brain, followed by STICKY graph model. SF-GD graph model is based on the hypothesis of evolution by duplications and divergence of the genes which produce proteins [26]. It reproduces the topological properties of the protein-protein interaction networks (PIN) with noticeable accuracy. Our experimental results also showed that the STICKY model has high GDD agreement, high Pearson correlation coefficients of the degree distributions, and low RGF distance and average shortest path length difference ratio when compared with SF-GD graph model. The STICKY model was also widely used in the investigation of protein-protein interaction networks, and the stickiness index can essentially capture the abundance and popularity of binding domains on a protein.

Since SF-GD and STICKY are not in conflict [27], we can also regard the stickiness as a property of the real brain network. In SF-GD model, networks revolute by duplication of nodes, and as a node is duplicated, it inherits most of the neighbors (interactions) of its parent nodes but gains some new neighbors as well [26]. Combined with stickiness index, a duplicated node would inherit its parents' stickiness index along with many of the parents' neighbors, and it would gain new neighbors in proportion to its inherited stickiness index and stickiness indices of the nodes already in the network [27]. The characteristics of “duplication,” “divergence,” and “stickiness” may explain the mechanism of structural brain network from biologically important properties shaped by natural selection. The “duplication” and “stickiness” make sure that the new neurons are created and contain both the functional and structural features. On the other hand, the “divergence” ensures that the new neurons have new connectivities with other neurons in different regions in order to enhance the cooperation and coordination ability of different regions in the human brain. This provides explanation why both STICKY and SF-GD models are suitable for describing the human brain networks; that is, the human brain networks are similar to SF-GD model and have STICKY property at the same time.

## Figures and Tables

**Table 1:** Average RGF distance in different groups.

Nodes	ER	ERDD	GEO	GEOGD	SF	SFGD	STICKY
Adolescent	3.13 ± 0.29	1.85 ± 0.21	1.39 ± 0.10	1.40 ± 0.09	1.05 ± 0.10	1.18 ± 0.18	0.95 ± 0.19
Adult	3.03 ± 0.29	1.80 ± 0.21	1.43 ± 0.10	1.45 ± 0.10	0.98 ± 0.11	1.07 ± 0.19	0.95 ± 0.18
Elderly	2.93 ± 0.22	1.79 ± 0.19	1.43 ± 0.09	1.47 ± 0.11	0.91 ± 0.10	0.98 ± 0.16	0.98 ± 0.17

**Table 2:** Average GDD agreement in different groups.

Nodes	ER	ERDD	GEO	GEOGD	SF	SFGD	STICKY
Adolescent	0.71 ± 0.04	0.71 ± 0.04	0.69 ± 0.04	0.62 ± 0.02	0.71 ± 0.04	0.67 ± 0.04	0.68 ± 0.04
Adult	0.71 ± 0.04	0.71 ± 0.04	0.69 ± 0.04	0.62 ± 0.03	0.71 ± 0.04	0.67 ± 0.04	0.68 ± 0.04
Elderly	0.72 ± 0.06	0.71 ± 0.05	0.70 ± 0.05	0.61 ± 0.03	0.72 ± 0.06	0.66 ± 0.05	0.68 ± 0.05

**Table 3:** Average Pearson correlation coefficients of degree distributions in different groups.

Nodes	ER	ERDD	GEO	GEOGD	SF	SFGD	STICKY
Adolescent	0.17 ± 0.20	0.97 ± 0.05	0.35 ± 0.10	0.27 ± 0.07	−0.04 ± 0.11	0.43 ± 0.08	0.52 ± 0.08
Adult	0.18 ± 0.17	0.98 ± 0.03	0.35 ± 0.10	0.25 ± 0.07	−0.08 ± 0.09	0.43 ± 0.09	0.51 ± 0.09
Elderly	0.16 ± 0.15	0.98 ± 0.05	0.35 ± 0.08	0.22 ± 0.04	−0.10 ± 0.09	0.43 ± 0.08	0.49 ± 0.08

**Table 4:** Average shortest path length difference ratio in different groups.

Nodes	ER	ERDD	GEO	GEOGD	SF	SFGD	STICKY
Adolescent	0.13 ± 0.02	0.13 ± 0.02	0.19 ± 0.02	0.30 ± 0.02	0.12 ± 0.02	0.06 ± 0.02	0.09 ± 0.01
Adult	0.13 ± 0.02	0.12 ± 0.02	0.19 ± 0.03	0.30 ± 0.03	0.12 ± 0.02	0.06 ± 0.02	0.09 ± 0.02
Elderly	0.12 ± 0.01	0.12 ± 0.01	0.20 ± 0.02	0.31 ± 0.03	0.10 ± 0.01	0.05 ± 0.02	0.08 ± 0.01

**Table 5:** Average clustering coefficient difference ratio in different groups.

Nodes	ER	ERDD	GEO	GEOGD	SF	SFGD	STICKY
Adolescent	0.74 ± 0.03	0.53 ± 0.05	0.33 ± 0.06	0.33 ± 0.06	0.57 ± 0.04	0.38 ± 0.07	0.52 ± 0.05
Adult	0.73 ± 0.03	0.52 ± 0.05	0.38 ± 0.05	0.37 ± 0.05	0.55 ± 0.03	0.34 ± 0.07	0.51 ± 0.05
Elderly	0.72 ± 0.02	0.53 ± 0.04	0.40 ± 0.05	0.39 ± 0.05	0.54 ± 0.03	0.32 ± 0.03	0.52 ± 0.04
